# AI-Driven Innovations in Pediatric Dentistry: Enhancing Care and Improving Outcome

**DOI:** 10.7759/cureus.69250

**Published:** 2024-09-12

**Authors:** Nawaf Alharbi, Adel S Alharbi

**Affiliations:** 1 Medicine, Riyadh Elm University, Riyadh, SAU; 2 Pediatrics, Prince Sultan Military Medical City, Ministry of Defense, Riyadh, SAU

**Keywords:** artificial intelligence, behavior management, neural network, oral health outcomes, pediatric dentistry

## Abstract

Artificial intelligence (AI) is transforming pediatric dentistry by enhancing diagnostic accuracy, streamlining treatment planning, and improving behavior management. This review explores current AI applications in detecting dental anomalies, categorizing fissure sealants, assessing chronological age, and managing patient behavior. The review also identifies emerging trends and future directions in AI technology that promise to further revolutionize pediatric dental care. By synthesizing recent research and clinical studies, this review aimed to inform dental professionals and researchers about the potential of AI to address traditional challenges and improve oral health outcomes for children.

## Introduction and background

Artificial intelligence (AI) has increasingly become an integral part of modern healthcare, with applications spanning various specialties, including pediatric dentistry [1,2]. AI, in simple terms, refers to the use of computers and algorithms to perform tasks that typically require human intelligence, such as decision-making, learning, and pattern recognition. In dentistry, AI has already shown its value in several key areas, including image-based automatic disease detection, diagnosis-support systems, and image segmentation for detecting oral traits [3,4]. For example, AI can assist in the early detection of dental caries or oral cancers through the analysis of radiographs, improving diagnostic accuracy and reducing human error. Additionally, AI techniques are being used to enhance the resolution of dentistry-related images, allowing for more precise treatment planning [5]. Robotics is also emerging as a promising area, with robotic assistance making complex dental procedures more efficient and less invasive [6]. These advancements are part of a larger shift toward digital dentistry, where AI plays a critical role in transforming clinical practice.

The current surge in AI applications in biomedicine, including pediatric dentistry, is driven by several factors. One major reason is the exponential growth in data collection over the past few decades. However, data alone is not sufficient; the ability to analyze and extract meaningful insights from this data has become essential. This is where AI, particularly machine learning (ML), comes into play. ML is a subset of AI that uses statistical models and algorithms to identify patterns within large datasets, enabling predictive analysis and decision-making based on the data [7,8]. For instance, in pediatric dentistry, ML can help predict which children are at higher risk for developing certain dental conditions based on their medical history, genetic factors, and lifestyle.

Deep learning (DL), a more advanced form of ML, has further revolutionized AI's applications in healthcare. DL mimics the way the human brain processes information by using artificial neural networks (ANNs), which consist of layers of interconnected nodes (or "neurons") that work together to analyze data and recognize patterns [9]. A specific type of DL, known as convolutional neural networks (CNNs), has successfully processed images. In dentistry, CNNs are being used to analyze 2D and 3D images, aiding in the design of dental prostheses and improving treatment outcomes [10,11]. These technologies are now being adapted to pediatric dentistry to create customized treatment plans that cater to the unique needs of children.

In pediatric dentistry, AI addresses several traditional challenges, such as improving diagnostic accuracy and enhancing treatment planning for young patients. Pediatric dentistry often involves managing the behavior of anxious or uncooperative children during dental visits. AI-driven virtual reality (VR) and augmented reality (AR) technologies are now being explored to create more engaging and less intimidating dental experiences for children, helping to manage their behavior and reduce dental anxiety [12]. Additionally, AI-powered tools can provide personalized oral health recommendations and preventive care strategies based on individual risk factors, which can be especially beneficial in pediatric settings [4].

Given the growing focus on integrating AI into dental practices, this review explores the impact of AI on pediatric dentistry, particularly its role in enhancing diagnostic accuracy, treatment planning, and behavior management. By addressing the current state of AI applications in pediatric dentistry, we aimed to provide dental professionals with insights into the potential of AI to overcome traditional challenges and promote innovations that improve oral health outcomes for children.

## Review

AI classification

In the context of wound care, data interpretation from patients' data, images, and videos represents the cornerstone for AI applications. As elaborated above, wound assessment (in terms of size, depth, wound bed, etc.) is challenging, and the current practice relies so far on subjective tools. Hence, AI technologies that can be trained to analyze wound images and correlate them with patients' data are much needed to fill the wound assessment and monitoring gap. Below, we briefly discussed AI technologies that interpret data from medical records or images.

ML, a statistical technique for fitting models to data, stands as a cornerstone in AI applications, particularly in healthcare. A significant application of ML is in precision medicine, predicting treatment outcomes based on patient data [13]. DL, an advanced form of ML, employs multiple layers of variables or features to predict outcomes, with applications in recognizing lesions in images, particularly through the application of convolutional neural networks (CNNs) [14]. The "deep" in DL refers to the multiple layers through which data is processed, allowing the system to learn complex patterns and features from large datasets [15].

CNNs, introduced by Yann LeCun in the 1980s with the development of the LeNET algorithm for automated handwriting recognition, have since become a cornerstone in pattern recognition tasks. CNNs draw inspiration from the human visual cortex, mimicking the way radiologists learn to interpret images by correlating their observations with known outcomes [16]. This analogy extends to CNNs' requirement for extensive labeled training data, allowing these networks to adjust internal weights and filters to improve recognition accuracy iteratively [17]. The convolutional layer is at the heart of a CNN, where input images are convolved with various weights to produce a series of filtered images. This is followed by pooling, which reduces the data's dimensionality by summarizing the filtered images' features and applying a rectified linear unit (ReLU) to introduce non-linearity by eliminating negative values [18].

The architecture of CNNs involves stacking multiple convolutional, pooling, and ReLU layers - referred to as "deep stacking" - to process data through increasingly refined layers [19]. Each iteration aimed to extract more abstract features, making the network capable of handling complex image recognition tasks. The culmination of this process is a fully connected layer, where the network makes predictions based on the learned features [20]. Errors in predictions lead to adjustments in the network through backpropagation, a method allowing the system to "learn from its mistakes" by optimizing the weights in response to the error gradient relative to the correct output [21].

AI application in pediatric dentistry

Dental Plaque Detection

Human mouths are home to around 700 different types of microbes [22]. Saliva contains microorganisms that stick to teeth, causing dental plaque to develop [23]. Dental plaque is the first step in many dental health conditions, including periodontitis, gingivitis, and caries. According to epidemiology studies, dental plaque frequently causes gingivitis in infants and adolescents, with various degrees of severity [24]. Childhood gingival disorders have the potential to progress and impact the periodontium in later life. To stop the disease from getting worse and to have the best possible treatment results, gingival disorders must be identified and diagnosed as soon as possible [25].

It is difficult to identify them, even for a skilled dentist, particularly in small amounts when it is difficult to differentiate between the tooth and the plaque. Clinicians have been marking the affected area with an explorer or a revealing solution until recently, but these techniques are cumbersome and not very convenient. They also have an unappealing flavor and leave stains on the mouth membrane, which are more aesthetic drawbacks [26]. Although there are additional techniques that use autofluorescence spectroscopy and digital imaging analysis, they do have financial and technological drawbacks [27]. The first imaging method to measure the entire area affected by a plaque was introduced with the advent of digital cameras and image analysis software [28].

DL techniques based on AI models are currently being investigated to determine primary teeth afflicted by plaque. With the help of 886 dental pictures, You et al., have effectively developed AI methods (CNN) that can identify the accumulation of plaque. The model's performance levels were clinically acceptable when compared to those of a pediatric dentist with training. Nonetheless, there are several drawbacks, including the outcomes differ significantly according to how accurate the final image is and it is unclear how the AI determines its strategies for identifying plaque [26].

A study by Yüksel et al. in 2023 used images of 168 teeth from 20 patients, ages 10-15 years, to evaluate the diagnostic precision of an AI system using DL to recognize dental plaque. They discovered that the AI system performed better in the detection of plaque, exhibiting 82% accuracy, 84% sensitivity, 83% F1 score, 87% recall, and 89% specificity [29]. Another AI model (YOLOv7 model) was used in a recent study conducted in 2024 by Lee et al. When it came to dental plaque detection in real-time, this AI model performed the best. Additionally, the associated app service offers a website-based administrative service that can be utilized to organize patient data and make it easier to generate findings from clinical data. This investigation proved the developed model's usefulness in assessing tooth plaque [30].

Oral Health Assessment Toolkits

Compared to other sections of the body, oral health is generally not given much emphasis by humans, and not even the majority of people receive an annual oral examination. This is particularly valid in developing and poor nations. To address these issues, the WHO created an oral health questionnaire for both adults and children. A research team's goal was to develop oral health evaluation toolkits that could accurately predict the children's oral health status index (COHSI) and referral for treatment needs (RFTN) by utilizing ML [31].

Based on the patient-reported oral health outcome measurement (Patient-Reported Outcome Measurement Information System {PROMIS} framework), Liu et al. developed a conceptual framework (oral health items bank system). Experts from the fields of pediatric dentistry, general dentistry, social science, and PROMIS developed the conceptual model for oral health. Physical, mental, and social health are the three fundamental elements that make up the conceptual paradigm. The oral health items bank system that this study develops serves as the basis for additional uses, like developing specialized brief forms for program assessment and dental policy development, among other uses [32].

To help parents assess their children's oral health condition and treatment needs, Wang et al. designed a toolkit that included a short form (SF) that defined health as having physical, mental, and social components. The way questions were phrased, the level of understanding of children and their parents, the time of day the survey was completed, and most importantly, the development of the ML algorithm all had a significant impact on the toolkit's (SF) accuracy. The toolkit was designed primarily to support the dentist during dental examinations, never to replace the actual inspection of the patient's mouth completely. The toolkit produced ranks based on the percentage among them, estimated the participants' overall dental health utilizing the COHSI rating, and determined whether or not the participants required treatment [31].

According to a study by Gajic et al., which used AI algorithms and statistical techniques to examine the relationship between dental health and teenage quality of life, both ML algorithms and human perception agreed on how the responses should be divided. AI algorithms have the potential to categorize the responses into various categories, providing access to information that would not be possible through a gender-based classification [33].

The adoption of Internet of Things (IoT) technology in pediatric dentistry by Adeghe et al. in 2024 offers a ground-breaking chance to transform early preventive and education approaches, encouraging proactive dental hygiene among kids. IoT can completely change the way dental care is provided and encourage lifelong practices for the best possible oral hygiene through real-time data collection, sophisticated analytics, and tailored interventions. To guarantee equal access and optimize the potential advantages of IoT in pediatric dental health, it is imperative to tackle ethical, privacy, and implementation obstacles, just like with any other developing technology [34].

Dentists, parents, and even children might utilize the ML-based toolkit findings to determine whether a patient needs dental treatment and to determine their current oral health status. To effectively address the need for clinical AI solutions, dental education must also promote digital literacy among future dental professionals. Therefore, applying machine learning to dentistry is quite advantageous since it allows us to obtain faster and more accurate outcomes [35,36].

Supernumerary Tooth Identification

Teeth over and above the average number of teeth are called supernumerary teeth. It is assumed that both hereditary and environmental factors have a role in the overabundance of teeth, even though the exact explanation is unknown [37]. It has been observed that the frequency of mesiodens is 0.1-7.0% [38].

Several issues, including diastema, crowding, resorption of the roots of nearby permanent teeth, dentigerous cysts, and problems with maxillary incisor eruption, can result from mesiodens. The difficulties of doing surgery will increase if inverted supernumerary teeth are not found early and are ignored, as they may migrate into the nasal cavity [39]. Consequently, it is essential to identify the supernumerary teeth beforehand and to intervene on time to avoid such issues.

The exact shape and detailed location data of the affected supernumerary teeth can be readily detected in three dimensions when cone-beam computed tomography (CBCT) is utilized for the diagnosis of supernumerary teeth [40]. Nonetheless, children are more radiosensitive than adults; thus, there is a significant risk of radiation exposure when kids' radiography is taken in the same settings as adults [41]. CBCT cannot be carried out regularly because of this. Before performing CBCT on children, the rationale for radiation exposure must be assessed. However, in contrast to CBCT, panoramic radiographs reveal the benefit of less radiation exposure and are frequently employed in dentistry since they can give a great deal of diagnostic information about the jaw with just one scan [42].

By using CNN models, AI is used to diagnose mesiodens [43]. The screening ability of young and fresh dental staff is primarily to blame for the absence of supernumerary teeth on panoramic radiography [44]. Furthermore, there is a lack of dentists who are skilled at identifying mixed dentition in kids. Notwithstanding these drawbacks, CNN-based DL might offer substantial assistance in the identification of supernumerary teeth [45]. Ahn et al. suggested that this approach could assist clinicians with little clinical experience in making more precise and quick diagnoses by using a DL model to identify supernumerary teeth in primary or mixed dentition. They used a variety of DL models, including Inception-ResNet-V2, ResNet 18, Squeeze Net, and ResNet 101, based on the straightforward hypothesis that deeper networks categorize mesiodens more accurately. When compared to human assessment, two deep learning models produced results noticeably faster, but their precision rate was marginally lower than that of human detection, which happened much faster [43].

Remarkably, all three CNN models - AlexNet, VGG16-TL, and InceptionV3-TL - performed admirably in a retrospective investigation by Mine et al. that identified the presence of extra teeth in the early mixed dentition phase. One advantage of these models is that they can be easily applied in a clinical context due to their simplicity. A few drawbacks exist, such as the scarcity of datasets and the capacity of AI-based models to categorize images that are beyond the grasp of two-dimensional panoramic radiography. A significant number of medical images that have been gathered from various facilities and institutions must be incorporated into their training to enhance their performance to a degree that is more practical in real-world settings. While there are certain drawbacks, such as restricted access to datasets from a single organization, AI-based models can identify images that are too small for two-dimensional panoramic radiography [45].

In 2024, Kim et al.'s study included 850 panoramic radiographs of pediatric patients (aged three to nine years). Supernumerary teeth in the upper anterior region were detected and segmented using the U-Net semantic segmentation technique. The trained model scored 91-92% accuracy and 94-95% F1-score in supernumerary teeth diagnosis, which was comparable with results from a human expert panel of 96%. When compared to human groups, the DL model's diagnostic length of 7.5 seconds was noticeably faster in the detection of supernumerary teeth [46].

Thus, the CNN-based DL approach is an intriguing technology that could help dentists with their diagnostic work; however, significant advancements in healthcare applications are needed before they can be put to use. Sooner rather than later, it will be necessary to build a comprehensive diagnostic tool that can handle a wider range of illnesses and ages. Therefore, the application of CNN-based DL could enhance screening performed by dentists who are not pediatricians and enable pediatric dentists to create treatment plans early (Table 1).

**Table 1:** The role of AI models in supernumerary tooth identification. *Names of databases or networks. CNNs: convolutional neural networks; NA: not available; AI: artificial intelligence

Studies	Algorithm architecture	Diagnostic tasks	Pre-trained network*	Sensitivity	Specificity	Accuracy	Precision	Recall	F1-score	Conclusions
Ahn et al. 2021 [43]	CNNs	Automatically classify mesiodens in primary or mixed dentition	SqueezeNet	NA	NA	0.833	0.779	0.960	0.855	Deep learning technologies have enabled more accurate and faster diagnoses
			ResNet-18	NA	NA	0.914	0.883	0.958	0.918	
			ResNet-101	NA	NA	0.927	0.911	0.948	0.928	
			Inception-ResNet-V2	NA	NA	0.924	0.916	0.934	0.925	
Mine et al. 2022 [45]	CNNs	Detecting the presence of supernumerary teeth during the early mixed dentition stage	AlexNet	0.825	0.780	0.805	NA	NA	NA	CNN-based deep learning shows promise for detecting supernumerary teeth during the early mixed dentition stage
			VGG16-TL	0.850	0.790	0.823	NA	NA	NA	
			InceptionV3-TL	0.833	0.780	0.809	NA	NA	NA	
Kim et al. 2022 [47]	CNNs	Identification of mesiodens in growing children/various dentition groups	Entire Panorama	NA	NA	0.839	0.840	0.848	0.844	The diagnostic performance of the deep learning system was based on the posterior molar space in panoramic radiographs, with potential for diagnosing various disorders automatically using only panoramic radiographs
			Manually Segmented ROIs	NA	NA	0.892	0.892	0.893	0.892	
			Automatically segmented ROIs	NA	NA	0.971	0.971	0.971	0.971	
Ha et al. 2021 [48]	CNNs	Identification of mesiodens in growing children/various dentition groups	YOLOv3	0.879	0.917	0.898	NA	NA	NA	The models are particularly effective in detecting mesiodens
Kuwada et al. 2020 [49]	CNNs	Classifying maxillary impacted supernumerary teeth	AlexNet	0.870	0.960	0.900	NA	NA	NA	These models could also classify the presence of impacted supernumerary teeth in the maxillary incisor region
			VGG-16	0.440	0.600	0.520	NA	NA	NA	
			DetectNet	0.920	1.000	0.960	NA	NA	NA	

Early Childhood Caries (ECC)

ECC is a complex condition because of the multiple elements that are related to it [50,51]. We question whether there is an intrinsic biological element, such as a hereditary factor, that has a bigger influence on caries formation because the causes of the problem appear to be unrelated to the behavioral and environmental variables. In a previous paper, the researchers used polymorphisms to predict the occurrence of dental caries using ANN. By implementing early treatments for dental caries and taking suitable steps, the data from such forecasts could help prevent cavities in children entirely and improve their overall quality of life [52].

According to Koopaie et al.'s findings, salivary cystatin S levels may be used to increase the efficacy of machine learning techniques for separating early childhood caries cases from caries-free controls. Instead of making it easier to identify important components for evaluating ECC levels, ML approaches let us create computer algorithms that can account for a variety of variables and their intricate relationships [53].

Research was done by Pang et al. to create a novel caries risk prediction model (CRPM) that considered genetic and environmental factors. CRPM can be utilized to recognize high-risk groups at the community level, enabling policymakers to schedule the appropriate preventive measures for the future [54].

Karhade et al. created and assessed an automated ML application for child categorization based on ECC. According to the study's findings, a parsimonious model performed best in terms of categorization. ECC risk may be predicted by a very naive ML model utilizing children's age and parents' perceptions of dental health. Additionally, ML can provide highly accurate classifications that can determine ECC status using demographic and proxy data [55].

Ramos-Gomez et al. proposed using a machine learning technique called Random Forest (RF) to select the most relevant questions from a parent questionnaire, aiming to predict the presence of active caries and generate the optimal set of questions. Based on these results, the group performed physical examinations of the research subjects [56]. In light of this, machine learning algorithms grounded in oral health surveys could help dentists predict dental caries in infants and young children. In addition to teaching patients and caregivers about the need for good oral hygiene, dental professionals can include the main indicators of dental caries in their caries risk evaluations.

An innovative use of AI in dentistry is the AICaries. With the use of AICaries, parents can seek treatment for their kids at an acute and reversible phase by using their smartphones to take pictures of their children's teeth and identify ECC. Additionally, parents can learn vital information on lowering their kids' risk of dental caries (Table 2) [57].

**Table 2:** The role of AI models in detecting ECC. *Names of databases or networks. ML: machine learning; ANNs: artificial neural networks; CNNs: convolutional neural networks; DC: dental caries; ECC: early childhood caries; AUC: area under the curve; PPV: positive predictive value; NA: not available; AI: artificial intelligence

Studies	Algorithm architecture	Diagnostic tasks	Pre-trained network*	Sensitivity	Specificity	Accuracy	Precision	Recall	F1-score	Conclusions
Park et al. 2021 [50]	ANNs	Predicting ECC	Logistic regression (final model)	0.799	0.531	0.765	NA	NA	NA	Can be useful in identifying high-risk groups and implementing preventive treatments
			XGBoost	0.769	0.581	0.763	NA	NA	NA	
			Random forest	0.759	0.400	0.755	NA	NA	NA	
			LightGBM	0.782	0.546	0.764	NA	NA	NA	
Zaorska et al. 2021 [52]	CNNs	Predicting dental caries based on chosen polymorphisms	Random regression	Null model-2 log-likelihood: 131.69; null model-2 log-likelihood: 131.69; p-value: <0.0001						The knowledge of potential risk status could be useful in designing oral hygiene and adopting healthy eating habits for patients
Koopaie et al. 2021 [53]	ANNs	Predicting ECC	Feed-forward neural network	1.000	0.721	0.909	NA	NA	NA	Considering clinical examination, demographic and socioeconomic factors, along with the salivary cystatin S levels could be useful for early diagnosis of ECC
			XGBoost	0.933	0.846	0.892	NA	NA	NA	
			Random Forest	0.933	0.769	0.857	NA	NA	NA	
			Support Vector Machine	0.866	0.846	0.854	NA	NA	NA	
Pang et al. 2021 [54]	ANNs	Caries risk prediction	Logistic regression	NA	NA	NA	NA	NA	NA	This is a powerful tool for identifying individuals at high caries risk at the community level
Karhade et al. 2021 [55]	ANNs	Classification of ECC	Logistic regression	NA	NA	NA	NA	NA	NA	This model is valuable for ECC screening
Ramos-Gomez et al. 2021 [56]	ANNs	Caries risk prediction	NA	0.940	0.680	0.710	NA	NA	NA	This model has the potential for screening DC
Wu et al. 2022 [58]	ANNs	Assessing the relation between ECC and oral microbes	NA	NA	NA	NA	NA	NA	NA	Further refinement is needed by considering more variables

Fissure Sealant Categorization

In most developed nations, dental caries affects 60-90% of school-age kids and a large portion of adults. It is a serious illness that negatively affects people's health [59]. Caries can worsen into pulp and periapical disease and might result in tooth loss if treatment is delayed. Pit and fissure sealing is a widely accepted and efficient way to prevent pit and fissure caries [60]. Fissure sealing and routine dental exams are crucial caries prevention techniques [61].

Dental sealants are frequently applied to prevent cavities on the chewing surfaces of molars. Dental restorations, sealants, and prosthodontic treatments are among the various interventions available for each type of dental disease that may be present. CNNs are extensively employed in categorizing images for diagnosis and the objective classification of diseased appearances; nevertheless, these networks require special training to recognize every issue. To help dentists, CNN is a crucial DL technique that uses massive amounts of data. Furthermore, as dental sealants are often white, they are easily recognized as the first line of treatment for a variety of dental issues. Therefore, the most sensible course of action would seem to be to fine-tune CNN to recognize dental sealants. A DL CNN was created by a research team under the direction of Schlickenrieder et al. to recognize these sealants from intraoral photos that may be read by a machine. When compared to the standard CNN-based classifications, this AI-based approach produced a high diagnostic accuracy. Before employing this AI-trained CNN in clinical applications, there were a few restrictions that required extensive dental study, repeated training for precise identification, and classification of the various disorders and associated repair processes [62].

A DL-based intelligent detection model (ToothNet) was created in 2024 by Xiong et al. The YOLOX framework was modified to create ToothNet, which allows for the simultaneous identification of fissure sealants and cavities. A total of 762 volunteers provided 1020 intraoral pictures. The suggested DL model performed well in identifying dental cavities and fissure sealants and was able to recognize many tasks simultaneously in intraoral pictures. The model is equivalent in detecting fissure sealants and has benefits in detecting cavities when compared to a dentist with one year of experience [63].

Chronological Age Assessment

Among the most accurate ways for assessing age is by dental age [64]. It is very helpful in fields like forensic medicine, endocrinology, orthodontics, pediatric dentistry, and anthropology [65,66]. The dental age evaluation can be utilized to establish the age of people who are adopting children from abroad, have memory loss, are undocumented immigrants, or lack identity documents [67].

One of two approaches that is typically used to analyze dental age is as follows: the panoramic method or the clinical method. The clinical approach yields very inaccurate findings even though it is simple to use and generates results quickly. However, orthopantomography techniques for evaluation, which measure tooth buds' mineralization, are more accurate [68]. Up until now, several techniques have been developed, each of which has demonstrated varying degrees of accuracy and may be applied to kids and teenagers of various ages.

In their study, Zaborowicz et al. combined digital pantomographic pictures and brain modeling to develop a novel technique for determining the chronological age of children and adolescents between the ages of four and 15 years. One of the main drawbacks of this approach is that it only uses pantomographic scans and does not utilize 2D photographs, while it is easier and has almost complete accuracy when it comes to metric age evaluation [68].

Zaborowicz et al. conducted a study utilizing three deep neural network models to identify the chronological age of children and adolescents between the ages of four and 15 years. The results demonstrated that neural modeling algorithms could precisely determine metric age utilizing proprietary tooth and bone markers [69]. Based on Demirjian's scores, Bunyarit et al. developed new dental maturity evaluations using ANN. They noticed that Malaysian Chinese children and teenagers' ages may be ascertained by the new dental maturity ratings [70].

In a fascinating work, Lee et al. used 18 radio morphometric characteristics that were taken from panoramic radiographs (PRs), with the main goal being the development of ML algorithms. They found that when compared to conventional estimates, ML algorithms are more effective at predicting age [71].

A study by Wu et al. demonstrated how well the AI-assisted dental age (DA) assessment identified the features of growth delay (GD) in children. The previous approaches showed overstated findings in both genders. However, the AI-assisted standards can produce significantly more accurate chronological age forecasts with average errors of less than 0.05 years. The ML models only showed delayed DA in the GD boys, whereas the CNN showed it in the GD youngsters of both genders [58].

A total of 144 baby skeletons, ranging in age from five months gestation to three years, were identified from the Granada osteological collection and included in the study by Martínez-Moreno et al. Their findings demonstrate the distinct benefits of applying ML techniques in comparison to conventional approaches. (1) These techniques reduce the assumed error; (2) they allow estimations to be made even in situations where teeth are missing, making them robust and applicable in a variety of situations; and (3) they allow the integration of qualitative and quantitative variables from multiple teeth, improving the accuracy of age estimation (Table 3) [72].

**Table 3:** The role of AI in chronological age assessment. ANNs: artificial neural networks; CNNs: convolutional neural networks; CA: chronological age; DA: dental age; NA: not available; AI: artificial intelligence

Studies	Algorithm architecture	Diagnostic tasks	Score	Difference between CA and DA in males	Difference between CA and DA in females	Difference	Accuracy	Conclusions
Zaborowicz et al. 2021 [68]	ANNs	Determining the chronological age	Radial basis function	NA	NA	NA	0.997	A new methodology for assessing chronological age using digital pantomographic images and a new set of tooth and bone parameters can be developed
Zaborowicz et al. 2022 [69]	CNNs	Estimating the age	Radial basis function	NA	NA	NA	0.96	The study shows that neural modeling approaches are effective tools for predicting metric age using proprietary tooth and bone indices
Bunyarit et al. 2020 [70]	ANNs	Dental age and chronological age estimation	Demirjian’s scores	-0.05±0.92	-0.06±1.11	Not statistically significant	NA	This methodology can be applied in both clinical and forensic cases

Deciduous and Young Permanent Tooth Detection

For recognizing objects, CNN is one of the most often used DL architectures. Pediatric patients' deciduous teeth are being assessed and counted more often with the use of DL techniques like CNN [73]. Various models, including R-CNN, Faster R-CNN, YOLOv3, and YOLOv4, have been applied to object recognition and detection tasks. The two types of object detection approaches are as follows: one-stage detectors (YOLO algorithm) and two-stage detectors (R-CNN, Mask-RCNN, and Faster R-CNN) [74].

Automated and sophisticated detection methods rely on tooth recognition to determine which teeth are affected by dental illnesses and to link those problems to the detected teeth. In the last 10 or so years, researchers have developed a variety of methods for classifying and numbering the teeth, moving away from CNN-based classification and toward region-based and threshold methods [73]. Nonetheless, in the area of automated tooth segmentation, CNN-based mapping has demonstrated higher precision [75]. In dentistry, digital diagnostic options that save time and effort are one step closer to being implemented with the utilization of panoramic radiographs to count primary teeth [73].

Caliskan et al. used CNN algorithms to detect and classify submerged molars, and they discovered that this method worked well. More research is necessary to determine whether a particular tooth germ is absent using tooth-numbering algorithms. To develop more precise treatment plans, dentists may find it useful to identify missing tooth germs [76]. Good sensitivity and accuracy scores were reported by Kılıc et al. when they looked into a quicker R-CNN inception v2 approach for primary tooth recognition and numbering on pediatric panoramic radiographs. They discovered from their research that only the primary teeth, which are also important for forensic identification, were found and counted [73].

Using YOLOv4, a CNN-based object identification model, Kaya et al. evaluated the efficacy of a DL system for automated tooth detection and counting. The model demonstrated the ability to identify and number both permanent and primary teeth. One popular one-stage detector model that can recognize and classify objects in a single image is the YOLOv4. This model is an object recognition system that operates in real-time, identifying multiple objects and representing the area of detection with bounding boxes around each object. YOLOv4's remarkable speed and accuracy made it the preferred choice for object detection. Two-stage detectors have been used in some research, with good object detection outcomes. Although two-stage detectors need more time and computation than one-stage detectors, they are frequently more accurate. As a result, YOLO serves as an illustration of a single-stage detector that can be used for quick and precise item classification. YOLO is distinct from previous CNN algorithms due to its capacity for real-time object recognition and its typical above-average performance across a wide variety of object classes [77].

Ectopic Eruption Detection

An ectopic eruption (EE) is when a tooth emerges in an unusual place; this usually happens in the early stages of mixed dentition. The most often missing tooth is the maxillary first permanent molar (PFM), with a reported incidence varying from 0.75% to 8.7% [49,78]. A localized eruptive disturbance that several circumstances could bring on is the EE of maxillary PFM. Potential reasons include dental anomalies like primary molar infra-occlusion, supernumerary teeth, big permanent first molar crown size, and greater mesial angulation of the eruptive path [3,79]. A few potential adverse outcomes are malocclusion, resorption of the distal surface of the primary second molar, loss of interdental space, and restriction of the dental arch [80]. Accordingly, an early diagnosis could help prevent unintended issues and help plan the course of treatment [81].

EE is identified by radiological and clinical examinations. Radiographs of various kinds, such as panoramic, occlusal, periapical, and CBCT, are employed in the diagnostic procedure. To ascertain tooth position and EE, all of these panoramic radiographs examine every tooth and the surrounding structures [82]. However, panoramic radiography has limitations, including restricted magnification, anatomical structural superimposition, and 2D imaging. AI and radiomics have developed quickly. Dentists can diagnose patients more thoroughly, consistently, and accurately with the use of multi-layered CNNs.

The model (nnU-Net) was more reliable and accurate in identifying and classifying EE in molars during the mixed dentition phase. A comparative study of the performance of U-Net, R2U-Net, attention U-Net, and nnU-Net models was conducted. The findings indicated that nnU-Net performed the best in terms of semantic augmentation [82]. The complexity and amount of the dataset affect the algorithm's performance, and semantic segmentation is challenging [83]. No-new-Net (nnU-Net) can provide the best model influence by dynamically adapting to any dataset by changing features, including information processing and training methods [84].

An automated screening method was created by Liu et al., which has accuracy comparable to that of pedodontists in identifying EE in maxillary molar. Although DL remains not 100% accurate when it comes to using them for identification, the authors discovered that AI-assisted recognition of photo models may increase the precision of human interpreters [85].

Behavior Management

AI for child behavior management uses the information and abilities they gained during dental school. Pediatric dentists are essential in diagnosing and treating dental conditions affecting children. However, modifying the child's behavior is frequently necessary to treat these illnesses safely and efficiently. It requires a range of interactions, with a focus on teaching and communication, between the patient, parent, dental team, and dentist. The objectives include reducing fear and anxiety and promoting awareness of the value of preserving optimal oral health and the steps required [86,87].

The field of pediatric dentistry has seen significant advancements in behavior management approaches in recent years. Innovative techniques have been added to traditional procedures to improve cooperation, lessen fear, and improve treatment outcomes. Non-pharmacological methods that have reduced anxiety and fostered trust include tell-show-do and positive reinforcement [88]. Furthermore, augmented reality (AR) and virtual reality (VR) technologies have become effective tools for engagement and distraction during dental procedures.

Techniques for sedation and anesthetic have also advanced to provide safe, customized care for children who are uneasy or reluctant. Practical behavior advice is enhanced by parent engagement and multidisciplinary cooperation with pediatricians and pediatric psychologists. Children are empowered to engage in oral health care when digital tools, interactive media, and instructional games are integrated, which enhances cooperation and promotes long-term dental health [89]. Together, these developments aim to create a welcoming and comfortable atmosphere that will support kids in developing a positive attitude about visiting the dentist for the rest of their lives.

Since it is their first time seeing a dentist, pediatric patients frequently arrive at the office extremely nervous. VR has arisen as a novel method that has been studied in the scientific literature to reduce patient tension and anxiety but to a limited degree. Several methods, including virtual reality exposure therapy (VRET) and in vivo exposure therapy (IVET), are used to treat anxiety. IVET, which is widely considered the gold standard method in this context, emphasizes exposing patients to their anxieties directly to lower anxiety levels. A more modern method called VRET uses computer-generated imagery to create scenarios that let patients experience their anxieties without having to deal with them in real life, which lowers anxiety [90]. A study looked at how virtual reality affected kids' ability to control their behavior [91]. According to the study, when patients used VR, their average anxiety and behavioral ratings were lower than those of the control group. Children find interactive and imaginative audiovisual representation in VR particularly appealing, which may be why these platforms are so helpful in lowering anxiety [92].

However, there are still disadvantages to employing AI in pediatric dentistry, especially in behavior management. These disadvantages include a lack of individualized connection, a limited ability to adjust to the behavior of the kid, a loss of human warmth and touch, as well as ethical and safety concerns [33,45].

Future direction

To fully realize the potential of AI in pediatric dentistry, several key advancements are necessary. First, more sophisticated AI algorithms must be developed to integrate and analyze multifaceted data from genetic, environmental, and lifestyle factors. This requires further research into how these diverse data sources interact with oral health outcomes in children, enabling the creation of more accurate and personalized risk assessments and treatment plans.

Additionally, practical steps should be taken to enhance the incorporation of AI into wearable devices and smart toothbrushes. This includes refining sensor technology and data integration systems to ensure continuous, non-invasive monitoring of oral health and providing real-time, actionable feedback to patients and caregivers. To support preventive care, these devices must be linked to AI-driven platforms that can predict and address oral health issues before they escalate.

In terms of patient experience, AI-driven virtual and augmented reality (VR and AR) applications need to be further developed to improve behavior management during dental visits. Research in this area should focus on how these technologies can be customized for children, reducing dental anxiety and improving engagement with oral health education.

For broader implementation, integrating AI with electronic health records (EHRs) and dental practice management systems must be prioritized. Research is needed to develop seamless, secure interfaces that streamline administrative tasks, automate patient communication, and enhance follow-up care. Collaboration between AI developers and dental professionals is essential to tailor these systems to the unique demands of pediatric dentistry.

Finally, AI has the potential to revolutionize pediatric dental research by analyzing large datasets to identify trends and predict outcomes. Future research should focus on developing AI tools capable of analyzing these datasets in real time, which could lead to the identification of novel treatment protocols and improved clinical decision-making. For effective implementation, AI technologies must become more accessible and affordable, which will require collaboration between industry leaders and healthcare policymakers to ensure widespread adoption in dental practices.

## Conclusions

AI is poised to revolutionize pediatric dentistry by offering innovative solutions to longstanding challenges in diagnosis, treatment, and patient management. By enhancing diagnostic accuracy, personalizing treatment plans, and improving patient experiences, AI can significantly improve children's oral health outcomes. The continued development and integration of AI technologies in pediatric dentistry will advance clinical practices and promote preventive care and early intervention, ultimately fostering better oral health for future generations.

AI models are very helpful both personally and in the community. They can detect and classify children into risk groups, identify and number teeth, diagnose early ectopic eruption, measure age, and more. Children can become more conscious of their dental health by using them as a tool in creating and evaluating school oral health programs. AI can be utilized as a supplemental tool in a controlled manner to maintain the human element and to reinforce the idea that pediatric and general dentists are the ones in charge of treatment protocols and decision-making. However, the time is not far off when AI and dentists could work together to improve patient care. As we look to the future, the ongoing collaboration between dental professionals, researchers, and technologists will be essential in harnessing the full potential of AI to transform pediatric dental care.
